# DEEP AGING: THE CONNECTION THAT SURVIVES DEATH AND ITS ROLE IN INCREASING HUMAN–NONHUMAN RELATIONSHIPS

**DOI:** 10.1093/geroni/igac059.1354

**Published:** 2022-12-20

**Authors:** Hong-Jae Park

**Affiliations:** Western Sydney University, Penrith, New South Wales, Australia

## Abstract

Ageing is part of life, and so is death. Although death will involve all of us over time, it is often regarded as a taboo topic, and bonds with the dead are seldom acknowledged in contemporary times. The aim of this paper is to present selected insights on the connection that survives death and its role in increasing human/non-human relationships, learned from two indigenous knowledges—whakapapa (genealogical connections in Maori) and filial piety (respect/care for parents and ancestors). Data were collected from semi-structured interviews with 49 key informants (Maori=25; Korean=24) between 2019 and 2021 in New Zealand and South Korea. A modified thematic analysis method was used to analyse the data obtained in a bilingual research context. The research findings show that the connectedness with ancestors or deceased loved ones is a significant part of the participants’ mental and social lives, emphasised in both whakapapa and filial piety/ancestor veneration traditions. Maori participants were likely to consider their natural environment (for example, land and water) as a common good for all generations, while Korean counterparts viewed it (for example, a mountain) as the place where ancestors were remembered and venerated. Participants’ awareness of the post-mortem relationships was associated with their connection with nature and spiritual practices. Overall, this study suggests that there are several possible ways that older people could do ‘something’ prior to death for their remaining families and friends, ranging from activities concerning death talk, end-of-life preparation, legacy building and after-life planning, to connectedness with nature and non-human beings.

